# Efficacy and safety of SQJZ herbal mixtures on nonmotor symptoms in Parkinson disease patients: Protocol for a randomized, double-blind, placebo-controlled trial

**DOI:** 10.1097/MD.0000000000008824

**Published:** 2017-12-15

**Authors:** Jing Shi, Jinzhou Tian, Ting Li, Bin Qin, Dongsheng Fan, Jingnian Ni, Mingqing Wei, Xuekai Zhang, Na Liu, Jianping Liu, Yumeng Li, Weiwei Liu, Yongyan Wang

**Affiliations:** a Institute of Neurodegenerative Diseases at Dongzhimen Hospital, Beijing University of Chinese Medicine; b Beijing Hospital; c Peking University Third Hospital; d Center for Evidence-Based Chinese Medicine, Beijing University of Chinese Medicine; e Consulting Center of Biomedical Statistics, The Academy of Military Medical Sciences; f Institute of Clinical Medicine, China Academy of Chinese Medical Sciences, Beijing, China.

**Keywords:** complementary therapy, nonmotor symptoms, Parkinson disease, randomized controlled trial

## Abstract

**Background:**

As a multisystemic neurodegenerative disorder, Parkinson disease (PD) has a broad spectrum of symptoms including motor and nonmotor symptoms (NMS). As shown in studies, NMS can also impact patient's quality of life, and many of them often go untreated. Chinese herbal medicines with multiconstituent may alleviate NMS in PD patients. This research is carried out to assess the efficacy and safety of a Chinese herbal formula for NMS, with its Chinese name acronym of SQJZ.

**Methods/design:**

It will be a multicenter, randomized, double-blind, placebo-controlled trial. Idiopathic PD with a Hoehn and Yahr scale score ≤4, aged 18 to 80 years, will be involved. About 240 patients will be randomly assigned to either SQJZ or placebo in a 2:1 ratio. There is a 2-week run-in period before the randomization, and the follow-up will be 24 weeks, including 12-week treatment period, with visit once every 4 weeks and 12-week washout follow-up. All participants are asked to maintain the regular medication schedule. SQJZ formula will consist of Chinese herbs with effects for insomnia, constipation, anxiety, and so on. The primary outcome will be measured using NMS scale, and secondary outcomes will include unified PD rating scale, PD sleep scale, the Parkinson fatigue scale, the constipation severity instrument, and PD Questionnaire-39. The primary efficacy analysis will be based on the intention-to-treat method, and mixed-model repeated-measures analyses will be used.

**Discussion:**

The findings from this research might provide evidence of the efficacy and safety of SQJZ Chinese herbal formula for treating NMS in PD patients. The results will sustain the broader use of SQJZ formula in PD.

## 1 Background

Parkinson disease (PD) is considered a multisystemic neurodegenerative disorder, together with the classic motor disability, and patients complain about a number of nonmotor symptoms (NMS).^[1–4]^ There is increasing recognition that the NMS of PD is extensive—more than 90% PD patients suffer from NMS.^[5,6]^ They have a significant negative relation with patients’ quality of life.^[6,7]^ NMS have underpinned this conceptual change that PD is a mixed motor, nonmotor, and multiorgan disorder rather than a pure movement disorder.^[8,9]^ The new criteria published for clinical and prodromal diagnosis of PD have incorporated a range of NMS. The PRIAMO study revealed that the most common NMS were fatigue (58%), anxiety (56%), leg pain (38%), insomnia (37%), urgency and nocturia (35%), drooling of saliva, and difficulties in maintaining concentration (31%).^[6]^ Several studies began to focus on NMS. A substudy of ADAGIO showed that rasagiline was associated with significantly less progression of fatigue compared with placebo over a 9-month period.^[10]^ The post hoc analysis of the RECOVER study suggested that rotigotine may benefit patients with sleep, pain, mood, and quality of life.^[11–13]^ However, the prospective study concluded that the improvement of multidomain nonmotor symptoms scale (NMSS) in rotigotine treatment was not superior to placebo.^[14]^ In general, both medicinal and nonmedicinal therapies^[15,16]^ are often advised for PD patients with NMS, but robust evidences for underpinning the clinical effects are limited.

Chinese herbal medicines have a long history used in traditional form for NMS in PD patients, but their real effects are uncertain. Some researchers showed that Chinese herbal medicines improved the NMS of constipation, hyperhidrosis, and sleep disorder.^[17–21]^ Some pilot study also showed that Chinese herbal extract could ameliorate gastroparesis and constipation in PD.^[21,22]^ However, there is still a lack of conclusive evidences to evaluate the efficacy and safety of Chinese herbal medicines by now.

We supposed Chinese herbal formula with multiconstituents may alleviate multiple symptoms at the same time. SQJZ formula, a mixture of Chinese herbs, was developed based on practical experiences from traditional Chinese medicine (TCM), which was responding to the leading symptoms of fatigue, insomnia, and constipation. According to the theory of TCM, the declined function of encephala and the increased toxin of phlegm turbidity are the 2 leading causes for the most manifestations of NMS in PD. Three principles of the prescription are reinforcing function of encephala, purging turbidity by dredging intestines, and tranquilizing mind.

The objective of this study is to assess the efficacy and safety of SQJZ versus placebo for NMS in a randomized clinical trial. As known, PD patients may experience NMS years before Parkinsonism onset,^[23–26]^ can SQJZ improving motor symptoms while improving NMS?

## 2 Methods/design

### 2.1 Study design

**Figure 1:**
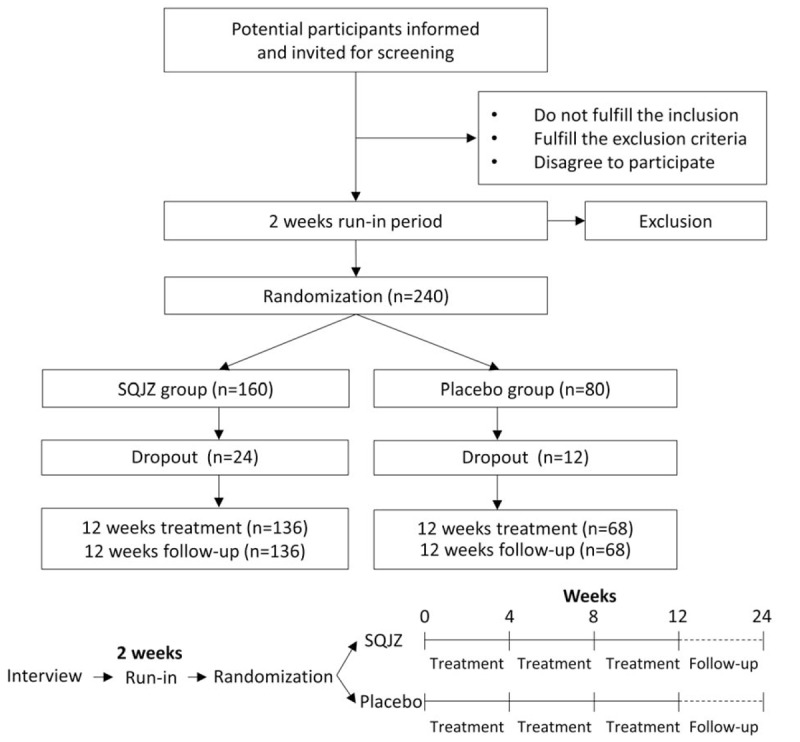
Study design flow chart. There is a 2-week run-in period before the randomization, and the follow-up will be 24 weeks, including 12-week treatment period with visit once every 4 weeks, and 12-week washout follow-up. Estimated 240 patients will be randomly assigned to either SQJZ or placebo in a 2:1 ratio.

This will be a multicenter, randomized, double-blind, placebo-controlled trial. Participants will be recruited from the PD outpatient of 3 hospitals in Beijing—Dongzhimen Hospital, Beijing Hospital, and Peking University Third Hospital. Patients will be recruited through poster and referral of general practitioners in community healthcare setting. There will be a 2-week run-in period before random allocation. Total research duration will be 24 weeks including 12-week washout follow-up. Participants will be followed up every 4 weeks, and the effect and the safety will be assessed at each visit (Fig. 1).

### 2.2 Ethics approval and consent to participate

The protocol has been approved by Ethics Committee of Dongzhimen Hospital affiliated to Beijing University of Chinese Medicine. Trial will be conducted under supervision of the clinical trials center in Dongzhimen Hospital. General management will follow Good Clinical Practice and Standard Operating Procedures. All data will be anonymized while participants must give written informed consent. Patients may leave this study at any time point without any constraint.

### 2.3 Selection and inclusion criteria

Inclusion criteria were as follows: (1) diagnosed as idiopathic PD according to Queen Square brain bank clinical diagnostic criteria^[27]^; (2) Hoehn and Yahr scale score ≤4; (3) male or female aged 18 to 80 years; (4) NMSS score ≥40^[28]^; (5) if receiving a stable dose of levodopa, anticholinergics, dopamine receptor agonists, monoamine oxidase B inhibitors, or amantadine, the subject should be maintained on a stable dose during the study period; (6) informed consent obtained.

Exclusion criteria were as follows: (1) receiving anti-PD drugs (eg, levodopa), but not under the guidance of professional doctors, either concurrently or within 28 days before the baseline visit; (2) receiving any of alpha-methyl dopa, metoclopramide, reserpine, sibelium, neuroleptics, monoamine oxidase-A inhibitors, methylphenidate, amphetamine; (3) receiving sedatives, hypnotics, selective serotonin reuptake inhibitors, anxiolytics, or other sleep-modifying medication unless with a stable dose at least 28 days before the baseline visit;(4) visual hallucination happened within 1 year after PD diagnosis; (5) delirium, epilepsy history, drug or alcoholabuse, serious disease history, or other conditions not suitable to be a participant; (6) laboratory abnormality, for example, serum creatinine ≥97 μmol/L, alanine amino transferase ≥40 U/L, aspartate amino transferase ≥40 U/L; (7) poor compliance; (8) participating in other clinical trials.

### 2.4 Intervention and control

All participants will take 2-week placebo in run-in period, then receive either treatment group of SQJZ formula or placebo in the next 12 weeks. SQJZ formula (Table 1), supplied by Beijing Tcmages Pharmaceutical Co., Ltd., will be packed in sealed opaque aluminum sachets with the direction outside, and will be administered twice daily orally by dissolving in 100 mL hot water. The placebo is made of SQJZ (5%) and dextrin (95%).^[29]^ It is also packed in sealed opaque aluminum sachets with the direction outside. Preparation process follow good manufacturing practice guideline to ensure all sourced raw materials, and intermediate and finished products strictly comply with standards of Chinese Pharmacopoeia 2010.

**Table 1:**
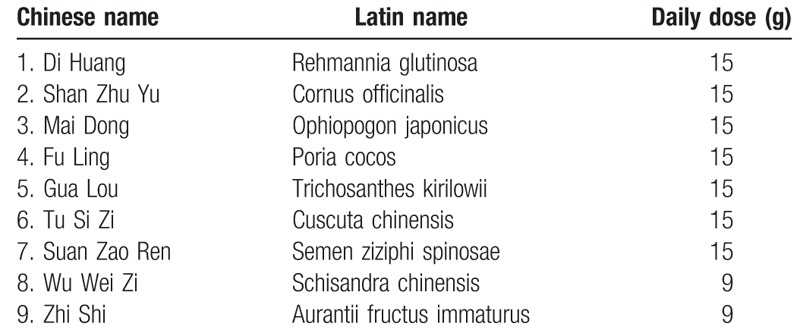
Primary composition of SQJZ formula.

Conventional western medicines will be maintained at a stable dose during the follow-up period. Glycerine enema will be allowed when participants have no defecation for 3 or more days. Other concomitant medication should also be recorded including the name, dose, and the reasons for use. To improve adherence, we will try to provide ongoing support to patients. Meanwhile, research assistants will give a call to remind the participants 1 week before the next visit, and a hotline will be set up to ensure the doctor–patient communication.

### 2.5 Randomization and masking

Patients will be assigned a study (patient) number after consenting to the study. Patients who meet all criteria for enrollment will be randomized to double-blind treatment in a 2:1 ratio. Assignment to treatment groups will be determined by a computer-generated random sequence. SQJZ and placebo is numbered in advance and will be dispensed in sequence. The placebo is made of SQJZ (5%) and dextrin (95%) to insure the mimic appearance, smell, and taste.^[29]^ Both researchers and participants will not know the assignment. The information on intervention assignments will be stored in the third consulting center of biomedical statistics.

### 2.6 Outcome measures and safety

The primary outcome will be NMSS, a validated tool for rating frequency and severity of NMS in PD.^[30]^ NMSS domains are as follows: cardiovascular, including falls; sleep/fatigue; mood/cognition; perceptual problems/hallucinations; attention/memory; gastrointestinal tract; urinary; sexual function; miscellaneous. NMSS use a 4-point scale to describe severity and frequency, and higher score indicates more severe conditions. The total NMSS score ranges from 0 to 350. Secondary endpoints include Unified Parkinson Disease Rating Scale (UPDRS),^[31]^ Parkinson Disease Questionnaire-39 (PDQ-39),^[32]^ Parkinson Disease Sleep Scale (PDSS),^[33]^ Parkinson Fatigue Scale (PFS-16),^[34]^ and the constipation severity instrument (CSI).^[35]^ The NMSS, UPDRS, PDSS, PFS-16, and CSI will be assessed at baseline, 4 weeks, 8 weeks, 12 weeks, and 24 weeks. Considering that the quality of life will not be changed in a matter of weeks, the PDQ-39 will be documented at baseline, 12 weeks, and 24 weeks.

During the course of the trial, safety will be assessed through laboratory testing and ECG. Laboratory testing will include liver function and renal function which herbal medicines may injure in the past reports. Participants will get safety assessment at baseline, 4 weeks, and 12 weeks. Investigators are responsible for monitoring the safety of patients who have entered this study. The occurrence and nature of pre-existing conditions including clinically significant signs and symptoms of the disease under treatment in the study will be recorded. Any unfavorable and unintended sign (including an abnormal laboratory finding), symptom, or disease after randomization will be reported as an adverse event, whether or not related to the investigational drug. Adverse events’ record will follow the WHO Adverse Reaction Terminology. Severe adverse events will be reported to Ethics Committee and principal investigator, and be assessed to determine whether further diagnostic investigation or treatment is warranted. If a severe adverse event occurs, appropriate treatment and follow-up will be given.

### 2.7 Data collection

Before initiation of the project, investigators from different branch center will participate in training to ensure the standard administration of data collection. The assessments of primary and secondary endpoints will be performed by 2 blinded investigators who are not involved in the randomization at the same time, and final scores will come from the average of the 2 investigators. If participant is unable to continue their face-to-face interview for any reason, we will collect as much information as possible and record it through making phone calls. To promote data quality, double data entry will be used.

### 2.8 Statistical analysis

The analysis of the primary variable will be based on the full analysis set: all randomized, treated patients who had a baseline and at least 1 postbaseline measurement for the primary variable. Only when the participant is not taking any drugs after randomization can he be ruled out. For missing value caused by dropout, the remaining date will use the last observation to carry forward. For missing value in the middle of complete cases, the most recent value of not missing date will be carried forward. Analyses of the safety data will be based on the safety set: all patients who were receiving at least 1 dose of trial medication.

Baseline demographic and clinical characteristics will be tabulated as means and standard deviations for continuous variables, and proportions and percentages for categorical variables. Standard baseline characteristics of sex, age, and medical history will be summarized for all patients. Group comparisons will be made using Fisher exact test for categorical data and analysis of variance, with independent factors for treatment of continuous data.

The primary objective of this study is to test the hypothesis that Chinese herbal formula SQJZ will benefit PD patients in alleviating NMS. Mixed-model repeated-measures analyses will be used to assess between-group differences in the modeled change in scores from baseline to 24 weeks. The change score from baseline at each visit will be the dependent variable. Independent variables in the model for fixed effects will be treatment (2 levels: SQJZ and placebo), visit (5 levels: baseline, week 4, week 8, week 12, and week 24), treatment-by-visit interaction, concomitant treatment (yes or no), baseline measures (UPDRS, NMSS), sex, age, and so on. An unstructured covariance matrix will be used to model the within-patient variance–covariance errors. If the unstructured covariance structure matrix results in a lack of convergence, the heterogeneous Toeplitz covariance structure followed by the heterogeneous autoregressive covariance structure will be used. The Kenward–Roger approximation will be used to estimate the denominator degrees of freedom. Similar methods will be used for assessment of secondary endpoints.

For safety analysis, the incidence of adverse events (symptoms, signs, diseases, laboratory tests, or abnormal electrocardiogram manifestations) will be tabulated. Chi-square test or Fisher exact test will be conducted for assessing the differences between SQJZ formula and placebo. All analyses will be performed using the software of Statistics Analysis System by a statistician, blinded to treatment allocation. The data of the trial will be reviewed every 6 months by an independent Data Safety and Monitoring Committee including a neurologist and a statistician, all of whom will be independent of the study.

### 2.9 Sample size

According to a previous study,^[14]^ we assumed the effect size of 0.4 in SQJZ treatment compared to placebo at 12 weeks, then a minimum of 204 patients (136 for SQJZ and 68 for placebo). In consideration of a dropout of about 15%, about 240 patients will be needed to achieve a sufficient statistical power of 85% at a significance level (alpha) of 0.05 in 2-tailed test.

### 2.10 Protocol amendments and dissemination

If there is any amendment to the protocol, approval must again be sought from the ethics committee before implementation. Participants who are randomized to placebo group will get a 12-week SQJZ and follow-up as post-trial compensation. Participants’ personal information will be confidentiality. Only relevant code will be sent to statistic analysis. The final dataset will be available to the principal investigator and the independent statistician. Data will be stored in a locked cabinet at the Dongzhimen Hospital Beijing University of Chinese Medicine, China, for 5 years.

Results will be published on the website of ClinicalTrials.gov in accordance with the CONSORT2010 Statement. We envisage the results will be published in high-impact generalist and specialist journals.

## 3 Discussion

Nonmotor symptoms are common in patients with established PD.^[1–4]^ The NMS of different race seems to be different. Recent memory (71.8%), constipation (68.2%), and attention dysfunction (62.4%) are the major NMS in Chinese PD patients, which shows great differences from western patients.^[36]^ Another study showed that NMS domains that influenced the PDQ-39 were sleep/fatigue, mood, gastrointestinal, urinary, and miscellaneous symptoms.^[37]^ These results strongly suggested that fatigue, sleep problems, and gastrointestinal symptoms play a key role in the life quality of PD patients. If there is a therapy directly aiming at these domains, it would be more helpful for Chinese PD patients.

The SQJZ formula is composited based on TCM practical experiences and TCM Ancient Books with the functions of Bushenjiannao (in Chinese, reinforcing kidney for better brain function), Ziyintongfu (in Chinese, nourishing Yin to treat constipation), and Qingreanshen (in Chinese, clearing interior heat to treat insomnia). It is believed to be suitable for dealing with complicated and diversified NMS, such as constipation, sleep disorder, and dizziness.^[22,38]^ There have been many clinical reports of Chinese herbal formula for treating NMS in PD patients, but disadvantages of trial design cannot underpin the long-term efficacy of TCM therapy. A good-quality controlled randomized clinical trial will bring credible evidence of efficacy and safety of SQJZ formula for treatment of NMS in PD patients.

This will be a study with restrictive inclusion, exclusion criteria, special instrument of NMS, and a clearly defined quality controlled intervention, and will be the first randomized controlled trial to evaluate the efficacy and safety of SQJZ formula in NMS of PD patients. Successful completion of this clinical trial may provide a new strategy in the treatment of PDNMS.
